# Editorial: Recent advancements in sleep homeostasis

**DOI:** 10.3389/fnins.2023.1231785

**Published:** 2023-06-13

**Authors:** Marcos G. Frank

**Affiliations:** Department of Neuroscience, Translational Medicine and Physiology, Elson S. Floyd College of Medicine, Washington State University Health Sciences Spokane, Spokane, WA, United States

**Keywords:** sleep, regulation, brain, autonomic, function

Sleep homeostasis is a regulatory process that increases sleep time and/or intensity as a function of prior time spent awake. It is considered a ubiquitous feature of sleep and is often considered a requirement for identifying sleep in the animal kingdom. In mammals, the accumulation and discharge of sleep drive have traditionally been measured with changes in the non-rapid-eye-movement (NREM) sleep electroencephalograph (EEG). More specifically, NREM EEG slow waves (at least in some frequency bands) increase and decrease in proportion to the time spent awake or asleep, respectively. This marker of sleep homeostasis has been observed in all mammals and most bird species. In animals that lack comparable forebrain areas (i.e., that lack a cerebral cortex or the equivalent), sleep homeostasis is usually identified by compensatory changes in sleep time following sleep loss. Sleep homeostasis has therefore been reported in highly diverse animal species, including insects, worms, cephalopods, and even jellyfish (Shaw and Franken, 2003; Anafi et al., 2019). Nevertheless, despite the central role sleep homeostasis plays in sleep biology, we still lack a complete understanding of its cellular, circuit, and molecular basis (for discussion, see Frank, 2021).

Having said that, this is a very exciting time to study sleep homeostasis. In the last 10–15 years, scientists have made important discoveries about its underlying mechanisms. For example, invertebrate studies have identified circuits and genes that when manipulated, alter sleep homeostasis (Anafi et al., 2019). Some of these mechanisms have analogs in mammals, which further focuses investigation. Studies in transgenic and inbred mice strains have further isolated signaling pathways that appear more related to sleep homeostasis than switching between brain states (Tafti and Franken, 2002; Franken and Dijk, 2009). Genome-wide-association-studies (GWAS), are beginning to isolate genetic contributions to differences in human sleep drive (Dashti et al., 2019). The role of glia in this process has also become a hot topic, as evidence continues to accumulate implicating these non-neuronal brain cells in sleep homeostasis (Ingiosi and Frank, 2022).

All of this makes this Research Topic *Recent advancements in sleep homeostasis* especially timely. The Research Topic includes articles spanning invertebrates and humans and addresses several key concepts. For example, our understanding of sleep homeostasis has been powerfully influenced by our understanding of circadian biology. As scientists, we strive for an elegant, molecular mechanism that can explain sleep drive in a manner comparable to our knowledge about biological clocks. Canonical “clock” genes have been identified as players in sleep homeostasis, but no simple molecular model of sleep homeostasis has emerged. This suggests that mechanisms identified in the canonical master clock [i.e., the suprachiasmatic nucleus (SCN) in mammals] may indeed overlap with sleep homeostasis, but not (so far) in an obvious way. It follows, therefore, that the more we know about different kinds of clocks, the better. In this vein, *the opinion article by* Rotinen is important because it addresses the molecular mechanisms governing peripheral (non-SCN) clocks.

As discussed above, invertebrate models have provided novel insights into sleep homeostasis. Jesús-Olmo et al. show the utility of this model by identifying the gene *pum* as a molecule that influences sleep homeostasis in flies. This is particularly interesting as *pum* mediates other homeostatic functions in the fly brain, including neuronal excitability. This Research Topic also includes new studies in rodent models. As shown by Choi et al., sex hormones influence sleep homeostasis as determined by classic methods of investigating these pathways (i.e., in mice hormonally intact, gonadectomized, or gonadectomized with hormone supplementation). Hung et al. explored the role of a canonical clock gene (*bmal1*) in basal sleep and wake expression in corticotropin-releasing factor (CRF) neurons. Previous studies have shown that CRF neurons communicate circadian signals, but the role of a canonical clock gene (*Bmal1*) in CRF neurons in basal sleep and wake had not been explored. Interestingly, the authors find that CRF neuron-specific Bmal1-deficient mice have normal sleep-wake organization, indicating that other mechanisms are likely involved in CRF-mediated effects on sleep expression. This Research Topic concludes with a study by Phillips et al., who provide new insights into interactions between signaling pathways implicated in sleep homeostasis (i.e., neurotrophins) and circadian regulation. This was accomplished by investigating sleep, compensatory changes in sleep after sleep deprivation, and circadian organization in mice with variations in Val66Met polymorphism.

In conclusion, sleep homeostasis remains one of the (many) mysteries about sleep. However, scientists have made progress in identifying some of the putative, underlying mechanisms. This has come about through a combination of approaches, including molecular and circuit neurobiology in diverse animal species.

## Author contributions

The author confirms being the sole contributor of this work and has approved it for publication.
